# Narrative Matters: Young people with lived experience discuss *I Swear*

**DOI:** 10.1111/camh.70082

**Published:** 2026-03-16

**Authors:** Jane Gilmour, Rayne Huggins

**Affiliations:** ^1^ The UCL Great Ormond Street Institute of Child Health, University College London London UK; ^2^ Great Ormond Street Hospital for Children NHS Foundation Trust London UK; ^3^ Tourette Action Champion UK

**Keywords:** Tourette syndrome, tics, Social Model of Disability, Medical Model, psychoeducation

## Abstract

Young people with lived experience of tics reflected on the recent film *I Swear*, a biography of John Davidson who grew up in the 1980s with Tourette syndrome. They considered the ways in which themes raised by the film connected with their own experiences. Their observations were discussed in the context of the Social Model of Disability and the Medical Model, illustrated by personal examples. The challenges of media representations and societal responses to tics in the recent past and in contemporary society were explored. They proposed that society and professionals must hold a position at the intersection of the Social Model of Disability and the Medical Model. The paper concluded with an urgent call for more extensive psychoeducation in the wider community to improve the quality of life for young people with tics.

This paper considers the utility of the Social Model of Disability (SMD) (Oliver & British Association of Social Workers, 1983) and the Medical Model in understanding the experience of young people with tics. *I Swear*, the recent film biography of John Davidson, invites us to reflect on societal attitudes to tic conditions in the recent past and raises issues pertinent for contemporary society. We meet John in 1983 in his first year of secondary school, popular and talented (scenes of young John's flirting with his classmates recall another Scottish coming of age film, *Gregory's Girl*), but with the gradual onset of increasingly complex phonic and motor tics, his life and that of his family unravels. We (JG, a clinical psychologist working with young people who have tics and RH, a peer nominated advocate for the tic community with lived experience) spoke to young people with tics about the film, the questions it poses, and the implications for society and professionals. Selected quotes from the young people who participated in the discussion are included in the paper.

Media and social media depictions of tic conditions typically ‘miss the mark’ according to one young person in the consultation group: an elegant and understated comment given the degree of misrepresentation in many cases. Often ‘creative licence’ generates a narrative in which tics are considered curious at best, comical at worst. Indeed, even an episode of a respected BBC documentary series, QED ‘John's Not Mad (1988)’ featuring the same John Davidson, characterises the condition with an ominous tone leaving the viewer more unnerved than informed. TS representation is peculiarly both lacking and inaccurate in society in comparison to other neurodevelopmental conditions such as Autism or ADHD. Tics have a lifetime prevalence of ~20% (see Scahill, Specht, & Page, 2014 for review); about the same number of children who wear braces in the UK. Why then is public understanding of, and empathy for, tics notably lagging so far behind other conditions? Our group considered media representations ‘the worst place for disability empowerment’. These characterizations inevitability impact mainstream understanding and ultimately the reactions of a local community. Uniquely—at least according to these young people—*I Swear* offered a relatable human experience that describes ‘what it's actually like…24/7’. ‘It felt empowering to watch’ a film that gets ‘under the skin’ of living with tics: ‘this is me, in a nutshell’.

Hollenbeck (Hollenbeck, 2003) argues that tics and Tourette Syndrome (TS) are ‘largely a disorder of the onlooker’. Our discussants connected with Hollenbeck's position and the film's thesis, which is broadly consistent with the Social Model of Disability (SMD) (Oliver & British Association of Social Workers, 1983). The framework proposes ‘an impairment’ does not cause ‘disability’, instead it is cultural attitudes that create disadvantage. The SMD was first described in the academic literature in the very same year that John's story starts in the film, and we witness his family, friends and numerous professionals reacting to tics and related behaviour with ignorance, prejudice or abuse. Perhaps we could assume that the principles emerging in academia at that time had yet to make an impact in mainstream society; nonetheless one heroic character in the film, Tommy the caretaker, maintains: ‘I don't think Tourettes is the problem, John…I think the problem is we don't know enough about Tourettes’.

Terzi's critique (Terzi, 2004) of the SMD acknowledges that it offers a crucial contribution to the debate on disability but questions the utility of the framework in explaining the *personal* challenges of lived experiences of many conditions. In our case, we argue that the SMD demands fundamental (and much needed) shifts in societal attitudes towards tic conditions, but it does not recognise the private experience of having tics; indeed we witness John's despair and depletion following tic episodes in many scenes of *I Swear*. One can accept that tics may be frustrating, exhausting or painful, but this must not invite a projected value on the person having these distressing experiences. Articulating the line between sympathy and empathy is relevant here (see Jeffrey, 2016 for review). Sympathy implies pity and is offered from a lofty position while empathy is an emotional bridge that connects us: an offer of compassion and understanding without any power inequality.

Have attitudes shifted since the dark days of 1983? Possibly. The young people discussing the film wondered if societal attitudes remain prejudicial but that it has become less acceptable to voice negative views overtly. ‘I don't think much has changed since 1983’. A comment justified having recently witnessed members of a cinema audience watching *I Swear* and laughing at scenes in which John's tics which were clearly causing him distress, unaware that members of the audience had tics themselves. An experience the young person describes as ‘disappointing’. Incidentally, opportunities to join and laugh *with* John are many and peppered throughout the film. In certain contexts, we are granted permission to see the humour in his experience, a stance which resonated with our discussion group: ‘I can laugh at my tics sometimes.’

John and another notable character in the film are both portrayed as having numerous complex vocal tics, including coprolalia (socially inappropriate or taboo words), giving uninformed viewers the strong indication that this is a core feature of the condition. While coprolalia is indeed part of John's portfolio of tics in life, it is relatively rare in the TS population, with recent estimates suggesting prevalence rates of ~5% of clinical samples (Nilles, Martino, Fletcher, & Pringsheim, 2023) and will be even less prevalent in other types of tic condition, given diagnostic criteria. Simple motor tics are the most common presentation in the TS population (see Scahill et al., 2014 for review), for example, an eye blink tic occurs in 57% of cases (Nilles et al., 2023). That the typical picture of TS was not represented in the film was one of the very few criticisms of *I Swear* in our consultation. Irritating but harmless, one might say. Yet one young person described their recent experience which illustrates how the media's fascination with coprolalia manifests in misunderstanding and negative preconceptions for the tic community in their day‐to‐day life. During a visit to the supermarket, a staff member asked the young person to be quiet during their tic episode, to which they patiently explained that they had Tourette syndrome. The employee responded: ‘you haven't got Tourettes at all… you aren't swearing’.

Though there was agreement in our discussion group that SMD had made significant contributions to evolving perceptions of disability and in developing policies to empower people with lived experience, the group did not dismiss the merits of the Medical Model (another example of developed and nuanced thinking in the reflection group). For example, many considered a diagnosis both emotionally and practically valuable. Following receiving their diagnosis, one young person said they could ‘breathe … because now I know what's going on’. Another noted that ‘it (the diagnosis) guides you towards a community’. They described the explanatory power of a diagnosis as a personal experience, noting there is no requirement to share it with anyone else: ‘it might just be for you’. Other members of the group remarked that when the diagnostic framework is shared with others, it unlocks resources as well as a common understanding in their educational and social spaces.

Evidence based behavioural tic management protocols (Habit Reversal Training and Exposure with Response Prevention) are effective in reducing—not eliminating—the frequency or intensity of tics but are indicated only on a case‐by‐case basis (Müller‐Vahl et al., 2022). A hardline SMD protagonist might argue that any intervention aimed at suppressing tics is oppressive and a reflection of societal judgement rather than an individual's clinical need. We maintain that the opportunity to *choose* to engage in a treatment that can offer agency over one's body (at least to some degree) is respectful and potentially empowering. This may be particularly important during adolescence when fulfilling autonomy is a fundamental developmental drive. Before offering any tic management intervention, it is crucial to establish why a young person is seeking treatment and explore if it is in response to pressure elsewhere in the system. The group we consulted observed that the decision to consider various treatment options, including drug regimes, was highly individual. ‘I wouldn't want to take medication but it's up to the person.’ Many of our discussants were interested in the most recent tic treatments being developed and delighted in the appearance of Barbara Morera, Chief Research Officer who played herself in the film. She introduced the *I Swear* audience to a promising non‐invasive neuromodulation clinical treatment (Maiquez et al., 2023)*;* one person noted ‘I liked the fact they added in the current research… it really informs people about what is going on right now’.

An account told with humanity and humour, *I Swear* offers a thought‐provoking commentary, documenting the experience of and reaction to difference. It provides powerful validation for the tic community, a group often neglected or misrepresented. Underscoring the message at the heart of the film, the young people we consulted propose that professionals and society hold a position at the intersection between the Social Model of Disability and the Medical Model. The ‘social’ must sit more prominently in the biopsychosocial explanation of tics, and with that, this paper makes an urgent call for more widespread psychoeducation in clinical and community settings. *Awareness* might just be the most effective intervention for many people with tics. It will certainly improve their quality of life; we swear.

## Conflict of interest statement

The author declares that there are no conflicts of interest in relation to this article.

## Funding information

This article was not supported by funding.

## Ethics statement

Ethics approval does not apply to this Narrative Matters article.

## Data Availability

Data sharing not applicable to this article as no datasets were generated or analysed for this Narrative Matters article.
